# Three Decades of Advances in Arabinogalactan-Protein Biosynthesis

**DOI:** 10.3389/fpls.2020.610377

**Published:** 2020-12-15

**Authors:** Jessy Silva, Ricardo Ferraz, Paul Dupree, Allan M. Showalter, Sílvia Coimbra

**Affiliations:** ^1^Departamento de Biologia, Faculdade de Ciências da Universidade do Porto, Porto, Portugal; ^2^LAQV Requimte, Sustainable Chemistry, Universidade do Porto, Porto, Portugal; ^3^Department of Biochemistry, University of Cambridge, Cambridge, United Kingdom; ^4^Department of Environmental and Plant Biology, Molecular and Cellular Biology Program, Ohio University, Athens, OH, United States

**Keywords:** arabinogalactan-proteins, arabinogalactan-proteinbiosynthesis, glypiation, proline hydroxylation, glycosylation, glycosyltransferases, hydroxyproline, cell wall

## Abstract

Arabinogalactan-proteins (AGPs) are a large, complex, and highly diverse class of heavily glycosylated proteins that belong to the family of cell wall hydroxyproline-rich glycoproteins. Approximately 90% of the molecules consist of arabinogalactan polysaccharides, which are composed of arabinose and galactose as major sugars and minor sugars such as glucuronic acid, fucose, and rhamnose. About half of the AGP family members contain a glycosylphosphatidylinositol (GPI) lipid anchor, which allows for an association with the outer leaflet of the plasma membrane. The mysterious AGP family has captivated the attention of plant biologists for several decades. This diverse family of glycoproteins is widely distributed in the plant kingdom, including many algae, where they play fundamental roles in growth and development processes. The journey of AGP biosynthesis begins with the assembly of amino acids into peptide chains of proteins. An N-terminal signal peptide directs AGPs toward the endoplasmic reticulum, where proline hydroxylation occurs and a GPI anchor may be added. GPI-anchored AGPs, as well as unanchored AGPs, are then transferred to the Golgi apparatus, where extensive glycosylation occurs by the action of a variety glycosyltransferase enzymes. Following glycosylation, AGPs are transported by secretory vesicles to the cell wall or to the extracellular face of the plasma membrane (in the case of GPI-anchored AGPs). GPI-anchored proteins can be released from the plasma membrane into the cell wall by phospholipases. In this review, we present an overview of the accumulated knowledge on AGP biosynthesis over the past three decades. Particular emphasis is placed on the glycosylation of AGPs as the sugar moiety is essential to their function. Recent genetics and genomics approaches have significantly contributed to a broader knowledge of AGP biosynthesis. However, many questions remain to be elucidated in the decades ahead.

## The Plant Cell Wall: the Protector of the Realm

Plant growth and development are essential processes that are regulated and supported by the cell wall (Serpe and Nothnagel, 1999). The plant cell wall is a dynamic and complex structure composed of different components that act together and contribute to cell wall architecture and function (Somerville et al., 2004). The cell wall plays different key roles in plant growth and development, cell differentiation, environmental sensing and signaling, intercellular communication, water movement, and defense against invading pathogens (Cosgrove, 2005; Jamet et al., 2008; Malinovsky et al., 2014; Bacete et al., 2018). Typically, the primary cell wall is a thin (0.1–1 μm) and flexible layer composed of three major classes of polysaccharides, namely cellulose, hemicelluloses, and pectins, along with cell wall proteins (CWPs; Somerville et al., 2004; Cosgrove, 2005; Atmodjo et al., 2013; Anderson and Kieber, 2020). Some specific cell types also include lignins (Jamet et al., 2006). Approximately 15% of the 27,000 *Arabidopsis thaliana* genes are involved in cell wall synthesis, modification, and turnover (Carpita et al., 2001; Wang et al., 2012).

Polysaccharides constitute up to 90–95% of the cell wall mass, whereas CWPs account for 5–10% (Cassab and Varner, 1988; Jamet et al., 2008). CWPs are dedicated to cell wall support, structure, signaling, and interactions with other proteins (Jamet et al., 2006). CWPs are classified into nine functional categories in *Arabidopsis*: (1) proteins acting on carbohydrates, which include glycoside hydrolases (GHs), glycosyltransferases (GTs), carbohydrate esterases, polysaccharide lyases, and expansins; (2) oxido-reductases such as peroxidases, multicopper oxidases, berberine bridge enzyme, and blue copper binding proteins; (3) proteases like serine carboxypeptidases and aspartic, cysteine, and serine proteases; (4) proteins with interaction domains, for example, lectins, leucine-rich repeat domains, and enzyme inhibitors; (5) proteins possibly involved in signaling, which include arabinogalactan-proteins (AGPs) and receptors; (6) structural proteins like glycine-rich proteins, extensins (EXTs), leucine-rich-repeat extensins, and proline-rich proteins (PRPs); (7) proteins related to lipid metabolism, for instance, glycine, aspartic acid, serine, leucine (GDSL) lipases and lipid transfer proteins; (8) miscellaneous proteins such as purple acid phosphatases, phosphate-induced proteins, and germin; and (9) unknown function proteins with domains of unknown function (DUFs; Showalter, 1993; Jamet et al., 2008; Albenne et al., 2013).

The cell wall hydroxyproline-rich glycoproteins (HRGPs) superfamily is subdivided into three families: lightly glycosylated PRPs, moderately glycosylated EXTs, and highly glycosylated AGPs (Showalter, 1993; Nothnagel, 1997; Showalter et al., 2010; Hijazi et al., 2014; Johnson et al., 2017b). HRGPs are extracellular intrinsically disordered proteins (IDPs) as they are rich in Pro, the most disorder-promoting residue due to its rigid conformation and have motifs that direct posttranslational modifications (Johnson et al., 2017a).

## Arabinogalactan-Proteins: All You Need is Sugar

AGPs are one of the most complex and diverse families of glycoproteins found in plants. AGPs may have a core-protein backbone rich in Pro/Hyp, Ala, Ser, and Thr (PAST) decorated by a diversity of carbohydrates (Showalter, 1993; Chasan, 1994; Schultz et al., 2002; Showalter et al., 2010). The amino acids of AGPs are often arranged in characteristic dipeptide repeats: Ala-Hyp, Ser-Hyp, Thr-Hyp, introduced as AG glycomodules (Tan et al., 2003; Ellis et al., 2010). The carbohydrate moiety of AGPs represents more than 90% of their total molecular mass, and it is composed mainly of arabinose (Ara) and galactose (Gal), as well as minor sugars such as glucuronic acid (GlcA), fucose (Fuc), rhamnose (Rha), and xylose (Xyl; Clarke et al., 1979; Fincher et al., 1983; Showalter, 1993; Chasan, 1994). AGPs have different molecular weights that reflect different extents of glycosylation of their specific protein cores. The extensive glycosylation of the protein backbone confers resistance to proteolysis (Showalter, 1993). Approximately half of the AGP family members are predicted to be tethered to the plasma membrane by a glycosylphosphatidylinositol (GPI) lipid anchor, being perfect candidates for signal perception and transduction (Youl et al., 1998; Oxley and Bacic, 1999; Sherrier et al., 1999; Svetek et al., 1999; Borner et al., 2002, 2003; Seifert and Roberts, 2007).

Positive reactions with β-Yariv reagent, a chemical reagent that specifically binds to the β-(1→3)-linked D-Galp backbone of AGPs (Yariv et al., 1967; Kitazawa et al., 2013), and immunolocalization studies with monoclonal antibodies that recognize AGP epitopes have shown that AGPs are ubiquitous in the plant kingdom, from bryophytes to angiosperms, and are also present in many algae (Clarke et al., 1979; Fincher et al., 1983; Showalter, 1993; Serpe and Nothnagel, 1999; Lee et al., 2005; Hervé et al., 2016). These glycoproteins were conserved during evolution, presumably because of their vital roles in plants (Serpe and Nothnagel, 1999). AGPs are found in plant cell walls, plasma membranes, apoplastic spaces, secretions, and intracellular multivesicular bodies (Herman and Lamb, 1992; Serpe and Nothnagel, 1999; Majewska-Sawka and Nothnagel, 2000; Ellis et al., 2010; Nguema-Ona et al., 2012).

AGPs are expressed in distinct cells and tissues and at particular stages of development. AGPs are a heterogenous family with their members, individually or collectively, implicated to function in many plant growth and development processes, such as cell proliferation and programed cell death, cell-cell signaling, embryo and postembryonic pattern formation, somatic embryogenesis, female and male gametophyte development, cell wall plasticizers, pollen tube growth and guidance, pollen incompatibility, root growth, xylem differentiation, secondary wall deposition, hormone signaling pathways, plant microbe interactions, and abiotic stress responses (reviewed in Showalter, 2001; Gaspar et al., 2004; Seifert and Roberts, 2007; Ellis et al., 2010; Nguema-Ona et al., 2013; Pereira et al., 2016). Recently, classical AGPs have been proposed to make a three-fold contribution: as a primary source of cytosolic Ca^2+^, as a pectic plasticizer and as Ca^2+^ signposts to the ovule (Lamport et al., 2018).

## Finding and Classifying AGPs: A History Overview

AGPs were initially discovered as polysaccharides isolated from suspension-cultured sycamore (*Acer pseudoplatanus* L.) cells (Aspinall et al., 1969). Since then, AGPs have fascinated and challenged researchers with their huge diversity of protein backbones discovered by genome sequencing. Two decades ago, the term AGP was defined as a group of molecules that presents three criteria (Clarke et al., 1979; Du et al., 1996; Knox, 1999): a core-protein backbone rich in Hyp; type II arabino-3,6-galactan polysaccharides (AGs); and the ability to bind to Yariv reagents (Yariv et al., 1962, 1967). However, since then, investigators have discovered that several AGPs do not fit these criteria (Du et al., 1996), as it is the case of AG-peptides from wheat (Fincher et al., 1974) and two glycoproteins from *Nicotiana alata* style that do not bind the Yariv reagent (Lind et al., 1994; Sommer-Knudsen et al., 1996). Moreover, other AGPs are histidine-rich (Kieliszewski et al., 1992) or have short oligoarabinosides (Qi et al., 1991).

AGPs cDNA were first isolated, cloned, and sequenced in 1994 from cultured pear (*Pyrus communis*) cells (Chen et al., 1994) and from *N. alata* styles (Du et al., 1994). AGPs were first classified based on the amino acid sequence composition as classical AGPs and non-classical AGPs (Mau et al., 1995; Du et al., 1996). Classical AGPs are characterized by the presence of an N-terminal hydrophobic secretion signal sequence, a central domain rich in PAST residues (including sites for Hyp-O-glycosylation) and potentially a hydrophobic C-terminal anchor addition sequence that directs the attachment of a GPI anchor (Du et al., 1996). Non-classical AGPs contain an N-terminal hydrophobic secretion signal sequence followed by one or more PAST-rich regions, which can be Hyp-O-glycosylated, along with other non-PAST rich regions, such as hydrophilic C-terminal Asn-rich domains (Mau et al., 1995; Du et al., 1996).

After the sequencing of the *Arabidopsis* genome in 2000 by the *Arabidopsis* Genome Initiative (AGI; Kaul et al., 2000), Borner et al. (2002) identified on the basis of sequence analysis 210 predicted GPI-anchored proteins and over 40% of these proteins had putative AG glycomodules, including 13 classical AGPs, 9 AG peptides, 18 fasciclin-like proteins, 8 phytocyanin-like proteins, 8 early nodulin-like proteins, and 9 lipid transfer protein-like proteins. In the same year, Schultz et al. (2002) conducted the first bioinformatics identification of AGPs based on sequence analysis, the amino acid bias method identified and classified 47 candidate AGP genes in four different classes: classical AGPs (13), Lys-rich AGPs (3), AG peptides (10), and fasciclin-like AGPs (FLAs; 21). The other classes are structurally similar to the classical AGPs but have different lengths and domains in their polypeptide core. AG peptides are short classical AGPs with only 10–13 amino acids (Schultz et al., 2000). Lys-rich AGPs contain a Lys-rich domain of approximately 16 amino acid residues between a Pro-rich domain and the C-terminus (Schultz et al., 2002; Sun et al., 2005). FLAs can have one or two fasciclin-like (FAS1) cell adhesion domains with 110–150 amino acids and one or two AGP domains (Gaspar et al., 2001; Schultz et al., 2002; Johnson et al., 2003). Then, Showalter et al. (2010) conducted a bioinformatics approach using the BIO OHIO program to identify HRGPs in the *Arabidopsis* genome based on the amino acid composition and specific motifs. This analysis allowed the identification of 166 HRGPs: 85 AGPs, 59 EXTs, 18 PRPs, and four hybrid AGP/EXTs (HAEs), which contain modules characteristic of AGPs and EXTs.

Nowadays, with the improvement of technologies, whole genome sequences of several plant species have been annotated, which allowed bioinformatic identification of AGPs and consequently, an update on AGPs classification. A bioinformatics approach named as finding-AGP based on AG glycosylation has been performed in 47 plant species from Chlorophyta to Eudicot, enabling the identification of thousands of putative AGPs. The number of AGPs varied between 48 in *Amborella trichopoda* and 313 in *Glycine max*, with 151 AGPs being identified in *Arabidopsis* (Ma et al., 2017). According to their polypeptide core and based on the presence/absence of particular motif/domains, AGPs are now classified into classical AGPs, AG peptides, Lys-rich AGPs, FLAs, early nodulin-like AGPs (ENODLs) with plastocyanin-like domains, xylogen-like AGPs (XYLPs) with non-specific lipid transfer protein (nsLTP) domains, other chimeric AGPs that do not belong in any of the mentioned groups and HAEs (Borner et al., 2002, 2003; Schultz et al., 2002; Johnson et al., 2003; Mashiguchi et al., 2009; Ma and Zhao, 2010; Showalter et al., 2010; Kobayashi et al., 2011; Ma et al., 2017). Therefore, we propose that AGPs should be defined as a large family of glycoproteins, which can share common features, such as the typical protein domain rich in PAST, the occurrence of Ala-Hyp, Ser-Hyp, and/or Thr-Hyp dipeptide repeats, the occurrence of an N-terminal hydrophobic secretion signal sequence, the presence of type II AGs attached to Hyp residues, the ability to interact with the synthetic chemical dye β-Yariv reagent (Yariv et al., 1962, 1967), which recognizes the β-1,3-Gal main chains of type II AGs (Kitazawa et al., 2013), the possibility to be attached to the plasma membrane by a C-terminal GPI anchor and the potential presence of other functional domains.

## AGP Biosynthesis Pathway: The Sugar Factory

**Figure 1:**
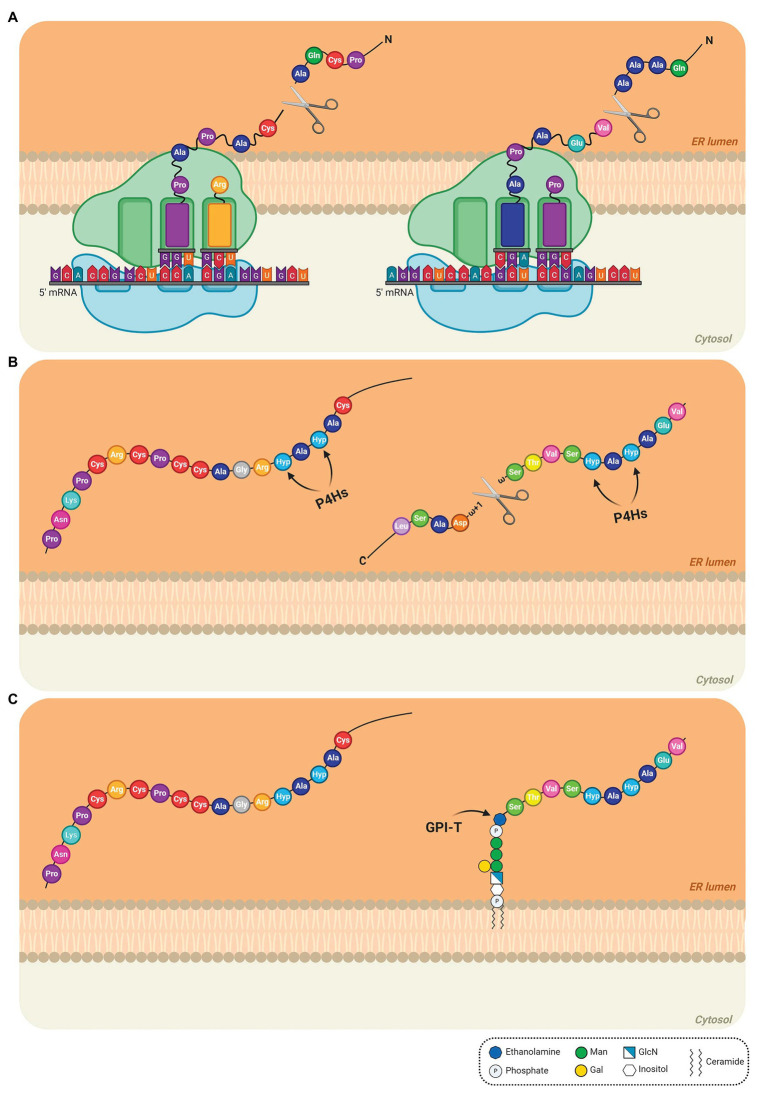
Detailed steps of the biosynthesis of AGPs in the endoplasmic reticulum (ER). **(A)** The N-terminal sequence is translated on the ribosomes, allowing the entry of the AGP into the ER. The N-terminal signal is removed and the AGP mRNA continues to be translated to produce the AGP protein backbone. **(B)** In the ER lumen, proline (Pro) residues are converted to hydroxyproline (Hyp) residues by prolyl-4-hydroxylases (P4Hs; Pro hydroxylation) and the C-terminal GPI anchor signal sequence is removed. The arrows indicate the site of action of P4Hs. **(C)** The preassembled GPI anchor is attached to the *ω* site of the mature protein *via* a transamidation reaction catalyzed by the transamidase complex (GPI-T). *AtAGP13* (At4g26320; NM_118765) and *AtAGP42* (At1g51915; NM_104072), two AG peptides were used as models in the schematic (left and right AGP, respectively). AtAGP13 (Q9STQ3-1) and AtAGP42 (Q8L9S8-1) have the smallest amino-acid sequence predicted to have or not a GPI anchor, respectively (Showalter et al., 2010). The extended Pro hydroxylation code was applied to determine, which Pro residues are hydroxylated (Canut et al., 2016; Duruflé et al., 2017). In these cases, only Pro residues after alanine (Ala) residues were converted to Hyp. Signal peptides and C-terminal anchor addition sequence positions were determined using UniProt (The UniProt Consortium, 2019). The GPI model structure was based on the GPI of PcAGP1 (Oxley and Bacic, 1999), which consists of phosphoethanolamine attached to the protein, three mannoses, one galactose, and glucosamine-inositol linked to phosphoceramide. Created by BioRender.com.

**Figure 2:**
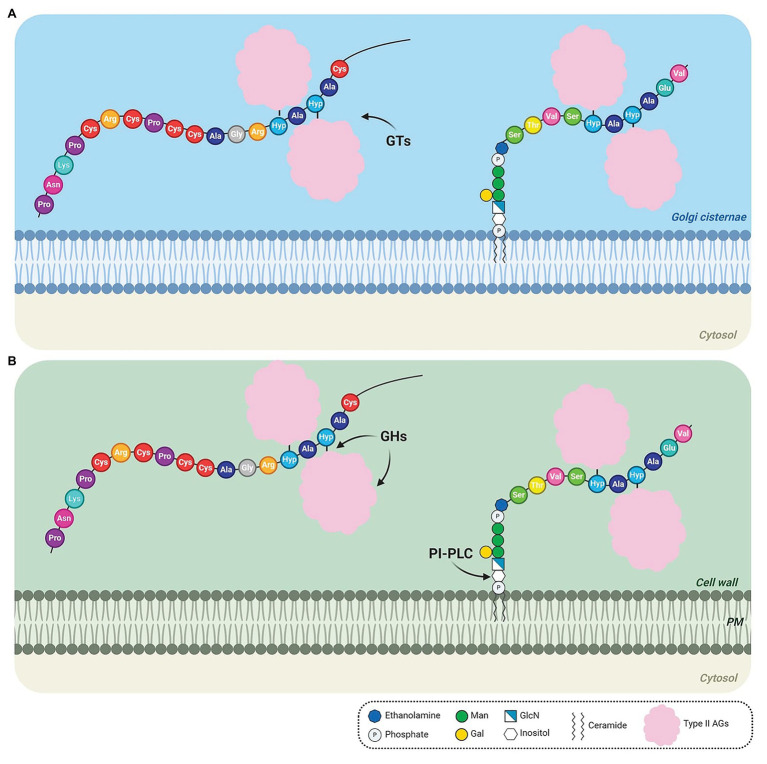
Detailed steps of the biosynthesis of AGPs after transport from the endoplasmic reticulum (ER). **(A)** AGPs are transported to the Golgi, where type II arabinogalactan polysaccharides (AGs) are O-glycosidically linked to hydroxyproline (Hyp) residues by glycosyltransferases (GTs). **(B)** O-glycosylated AGPs are transported *via* Golgi vesicles to the cell wall, where they remain temporarily attached to the outer leaflet of the plasma membrane (PM) in the case of GPI-anchored AGPs. GPI-anchored AGPs may be released from the PM by PI-PLC phospholipase. The AGs may be cleaved by glycoside hydrolases (GHs). The arrows indicate the site of action of GTs **(A)**, GHs and PI-PLC **(B)**. *AtAGP13* (At4g26320; NM_118765) and *AtAGP42* (At1g51915; NM_104072), two AG peptides were used as models in the schematic (left and right AGP, respectively). AtAGP13 (Q9STQ3-1) and AtAGP42 (Q8L9S8-1) have the smallest amino-acid sequence predicted to have or not a GPI anchor, respectively (Showalter et al., 2010). Based on the Hyp contiguity hypothesis, non-contiguous Hyp residues are arabinogalactosylated. The GPI model structure was based on the GPI of PcAGP1 (Oxley and Bacic, 1999), which consists of phosphoethanolamine attached to the protein, three mannoses, one galactose, and glucosamine-inositol linked to phosphoceramide. Created by BioRender.com.

The biosynthesis of AGPs comprises the addition and removal of amino acids, lipids, and carbohydrates. As some AGPs are composed of approximately 90% sugar, the AGP biosynthetic pathway resembles a candy factory occurring inside the cells. The production line involves sequential operations and begins with the translation of the N-terminal signal sequence of AGPs on ribosomes, which allows entry into the endoplasmic reticulum (ER) and endomembrane system (Figure 1A). Translation continues of the AGPs with concomitant removal of the signal peptide (Figure 1A) and hydroxylation of Pro residues by prolyl hydroxylase enzymes located in the ER (Walter and Johnson, 1994; Schatz and Dobberstein, 1996; Faye et al., 2005; Figure 1B). The resulting AGP backbone can be further modified in the ER if it contains a GPI anchor addition sequence (Schultz et al., 1998; Yeats et al., 2018; Figure 1C). Subsequently, AGPs, either free in the ER lumen or tethered to the ER membrane by a GPI anchor (Figure 1C), are transported to the Golgi apparatus to allow for the successive addition of various monosaccharide units to the protein backbone (Figure 2A). Several enzymes constitute the required machinery for these consecutive sugar additions, and once the process is finished, the final products are transported to the plasma membrane for direct release to the cell wall (if no GPI anchor is present) or immobilized to the outer leaflet of the plasma membrane *via* a GPI anchor, with the bulk of these AGPs occurring in the periplasmic space (Figure 2B). Subsequent proteolytic processing can cleave the GPI anchor and release the AGP to the cell wall (Schultz et al., 1998).

### Pro Hydroxylation: Getting Ready

The N-terminal signal sequence, recognized by the signal recognition particle, is proteolytically removed, directing AGPs into the ER, where extensive post-translational modifications begin (Walter and Johnson, 1994; Schatz and Dobberstein, 1996; Figure 1A). Pro hydroxylation is a prerequisite for O-glycosylation of CWPs on Hyp residues (Faye et al., 2005). Pro hydroxylation begins in the ER and selected Pro residues are converted to Hyp residues by a multigene family of enzymes, prolyl-4-hydroxylases (P4Hs) providing reactive hydroxyl groups for O-glycosylation (Faye et al., 2005; Yuasa et al., 2005; Hijazi et al., 2014; Nguema-Ona et al., 2014; Figure 1B). P4Hs (EC 1.14.11.2) belong to the family of 2-oxoglutarate-dependent dioxygenases and catalyze the formation of 4-Hyp requiring 2-oxoglutarate and O_2_ as co-substrates, Fe^2+^ as cofactor and ascorbate (Kivirikko and Myllyharju, 1998; Vlad et al., 2007). Several plant P4Hs have been partially characterized *in vitro* and *in vivo*: AtP4H1 (Hieta and Myllyharju, 2002; Asif et al., 2009), AtP4H2 (Tiainen et al., 2005; Velasquez et al., 2011b), AtP4H5 and AtP4H13 (Velasquez et al., 2011a,b 2015), NtP4H (Yuasa et al., 2005), DcP4H1 and DcP4H2 (Vlad et al., 2010), and CrP4H1 (Keskiaho et al., 2007). Only the activity of AtP4H1 and AtP4H2 from the 13 member P4H family in *Arabidopsis* has been fully characterized (Velasquez et al., 2015). Recent evidence indicates that P4Hs may form protein complexes required for Pro hydroxylation as AtP4H5 forms homodimers/heterodimers with AtP4H2 and AtP4H13 in the Golgi (Velasquez et al., 2015).

To date it remains difficult to predict with confidence, which Pro residues will be hydroxylated (Duruflé et al., 2017). The extended Pro hydroxylation code, based on the initial Pro hydroxylation rules (Kieliszewski and Lamport, 1994) and additional experimental LC-MS/MS and Edman sequencing data, indicates that Pro residues are typically hydroxylated when they occur after Ala, Gln, Hyp, Pro, Ser, Thr, and Val residues (Canut et al., 2016; Duruflé et al., 2017).

### Glypiation: GPI Anchoring

The addition of GPI-anchors, also referred to as glypiation, is a post-translational modification allowing AGPs to be attached to the plasma membrane (Duruflé et al., 2017). In *Arabidopsis*, 55 of the 85 identified AGPs are predicted to contain a GPI-anchor addition motif (Showalter et al., 2010). GPI-anchors allow attachment of proteins to the cell surface and may increase their lateral mobility in the membrane, exclusion from clathrin-coated pits, targeting to membrane microdomains/lipid rafts, and function in signal transduction pathways (Schultz et al., 1998; Ellis et al., 2010; Desnoyer and Palanivelu, 2020). The GPI-attachment signal (GAS) in the C-terminal region of the protein consists of ~11 polar residues, followed by the *ω* region of ~4 small residues containing the *ω* site, a spacer region of ~6 moderately polar residues and a C-terminal hydrophobic region of variable length between 9 and 24 residues (Schultz et al., 1998; Eisenhaber et al., 2003; Ellis et al., 2010; Desnoyer and Palanivelu, 2020). The highly conserved GPI moiety is initially synthesized on the cytosolic surface of the ER *via* the sequential addition of glucosamine, three α-linked mannosyl (Man) residues, and phosphoethanolamine to phosphatidylinositol (Schultz et al., 1998; Yeats et al., 2018; Beihammer et al., 2020; Desnoyer and Palanivelu, 2020). Although GPI anchor biosynthesis has not been biochemically studied in plants, the proteins that catalyze this pathway have been well studied in mammalian cells, yeast, and protozoa (Schultz et al., 1998; Pittet and Conzelmann, 2007; Morotti et al., 2017; Desnoyer and Palanivelu, 2020; Kinoshita, 2020). Nevertheless, this process may be conserved as orthologs of GPI biosynthetic genes are found in plant genomes (Schultz et al., 1998; Ellis et al., 2010; Desnoyer and Palanivelu, 2020).

In mammals, the first step involves the transfer of β-N-acetylglucosamine (GlcNAc), from the nucleotide sugar UDP-GlcNAc, to phosphatidylinositol (PI) to generate GlcNAc-PI by the GPI-N acetylglucosaminyltransferase (GPI-GnT) complex, which contains seven subunits (PIG-A, PIG-C, PIG-H, PIG-Q, PIG-P, PIG-Y, and DPM2). The product formed is de-N-acetylated subsequently by PIG-L, a GPI deacetylase, to yield GlcN-PI. At some point in the process, the synthesis switches from the cytoplasmic to the luminal face of the ER by an unknown flippase enzyme. In the ER lumen, GlcN-PI is acylated by the acyltransferase PIG-W to generate GlcN-(acyl)PI. Then, three Man residues, donated by dolichol-phospho-mannose (Dol-P-Man), are sequentially added to GlcN-(acyl)PI by the GPI-mannosyltransferases PIG-M, PIG-V, and PIG-B. The addition of phosphoethanolamine (PEtN) on the third Man residue is catalyzed by PIG-O and PIG-F (Schultz et al., 1998; Ellis et al., 2010; Beihammer et al., 2020; Desnoyer and Palanivelu, 2020; Kinoshita, 2020). The resulting GPI structure is ready to be transferred to proteins but other side chains may be added, such as phosphoethanolamines or sugars may be linked to Man and acyl groups linked to inositol (Luschnig and Seifert, 2010; Beihammer et al., 2020).

The GPI biosynthetic process converges with the cotranslational insertion of the protein backbone into the ER. The GAS is recognized and proteolytically cleaved between the *ω* and *ω* + 1 sites by the GPI transamidase (GPI-T) complex and the GPI anchor is attached to the *ω* site of the mature protein by a transamidation reaction (Schultz et al., 1998; Ellis et al., 2010; Yeats et al., 2018; Beihammer et al., 2020; Desnoyer and Palanivelu, 2020; Figure 1C). The mammal GPI-T consists of five subunits, PIG-K, GPAA1, PIG-S, PIG-T, and PIG-U (Kinoshita, 2020).

Some GPI biosynthesis enzymes in mammals have homologs already characterized in the *Arabidopsis* genome: SETH1 for PIG-C, SETH2 for PIG-A, PEANUT1 (PNT1) for PIG-M, PEANUT 5 (PNT5) for PIG-W, ABNORMAL POLLEN TUBE GUIDANCE1 (APTG1) for PIG-B, AtGPI8, and AtPIG-S (Lalanne et al., 2004; Gillmor et al., 2005; Dai et al., 2014; Bundy et al., 2016; Liu et al., 2016; Beihammer et al., 2020; Desnoyer et al., 2020; Desnoyer and Palanivelu, 2020). Although several different structures of GPI anchors have been observed in several kingdoms, to date, in plants, the only known structure was determined on PcAGP1 isolated from pear (Oxley and Bacic, 1999). This structure contains a glycan moiety conserved in all eukaryotic GPI anchors, D-Manα(1–2)-D-Manα(1–6)-D-Manα(1–4)-D-GlcN-inositol with a specific β(1–4)-galactosyl substitution of the 6-linked Man residue, it is devoid of PEtN side chains and contains a phosphoceramide, composed of phytosphingosine and tetracosanoic acid, instead of the common glycerolipid (Oxley and Bacic, 1999; Ellis et al., 2010; Yeats et al., 2018; Desnoyer and Palanivelu, 2020; Figure 1C). Moreover, an AGP isolated from *Rosa* sp. cell suspension cultures also included a ceramide, composed of tetracosanoic acid and 4-hydroxysphinganine, as the GPI lipid component (Svetek et al., 1999). These results suggest the PI glycerolipid moiety of some GPI-anchored proteins is remodeled to contain ceramide, as described in yeast (Bosson and Conzelmann, 2007). Discovering other plant GPI structures will identify possible forms of GPI and determine the conservation of these structures in plants (Desnoyer and Palanivelu, 2020).

### Glycosylation: Becoming Sweeter

AGPs free in the ER and AGPs anchored to the ER membrane are then transferred to the Golgi apparatus, where they undergo glycosylation (Figure 2A). Glycosylation is one of the major post-translational modifications found in almost every living organism. It is performed by GTs and includes N-glycosylation, O-glycosylation, and glypiation (Hurtado-Guerrero and Davies, 2012; Duruflé et al., 2017). Each of these post-translational modifications occurs on specific amino acid sequences (Duruflé et al., 2017). The addition of carbohydrates on a polypeptide backbone may affect the physico-chemical properties of a protein, including resistance to thermal denaturation, protection from proteolytic degradation, solubility, and it can alter essential biological functions (Faye et al., 2005).

N-glycosylation occurs on Asn residues in Asn-X-Ser/Thr specific sequences, where X can be any amino acid except Pro (Faye et al., 2005; Duruflé et al., 2017). N-glycosylation starts in the ER by the co-translational transfer of Glc_3_Man_9_GlcNAc_2_, an oligosaccharide precursor, onto the amide nitrogen of Asn residues. During the transportation of the glycoprotein along the secretory pathway, the N-glycan undergoes a maturation process that involves the removal and the addition of sugar residues in the ER and the Golgi (Faye et al., 2005; Nguema-Ona et al., 2014). Classical AGPs do not contain this conserved sequence but many chimeric AGPs, including FLAs, contain the consensus sequence for N-glycosylation (Du et al., 1996; Johnson et al., 2003; Ellis et al., 2010).

O-glycosylation is the most complex type of glycosylation and, in plants, it occurs predominantly on Hyp residues and less often on Ser and Thr residues in the Golgi apparatus (Nothnagel, 1997; Faye et al., 2005; Duruflé et al., 2017). The Golgi apparatus, the central organelle in the secretory pathway, is responsible for glycosylation, protein sorting, and secretion; it contains a diverse group of membrane-bound GTs required for synthesis of a variety of linkage types (Nikolovski et al., 2012). O-glycosylation of Hyp is a complex mechanism unique to plants that involves the transfer of a glycan from the donor substrate to the acceptor hydroxyl group of Hyp residues (Faye et al., 2005). Gal can be linked to Ser and Hyp residues, whereas Ara can only be linked to Hyp residues (Duruflé et al., 2017). HRGPs are glycosylated by two types of O-glycosylation on their Hyp residues in the Golgi apparatus: Hyp arabinosylation and Hyp arabinogalactosylation (Kieliszewski, 2001; Nguema-Ona et al., 2014). The Hyp contiguity hypothesis (Kieliszewski and Lamport, 1994) predicts that contiguous Hyp residues are arabinosylated, adding short 4-6 residue long oligoarabinoside chains, as occurs in EXTs, whereas non-contiguous Hyp residues are arabinogalactosylated, adding larger 30–150 residue acidic or neutral AGs polysaccharides, as occurs in AGPs (Shpak et al., 1999, 2001; Kieliszewski, 2001; Faye et al., 2005).

**Figure 3:**
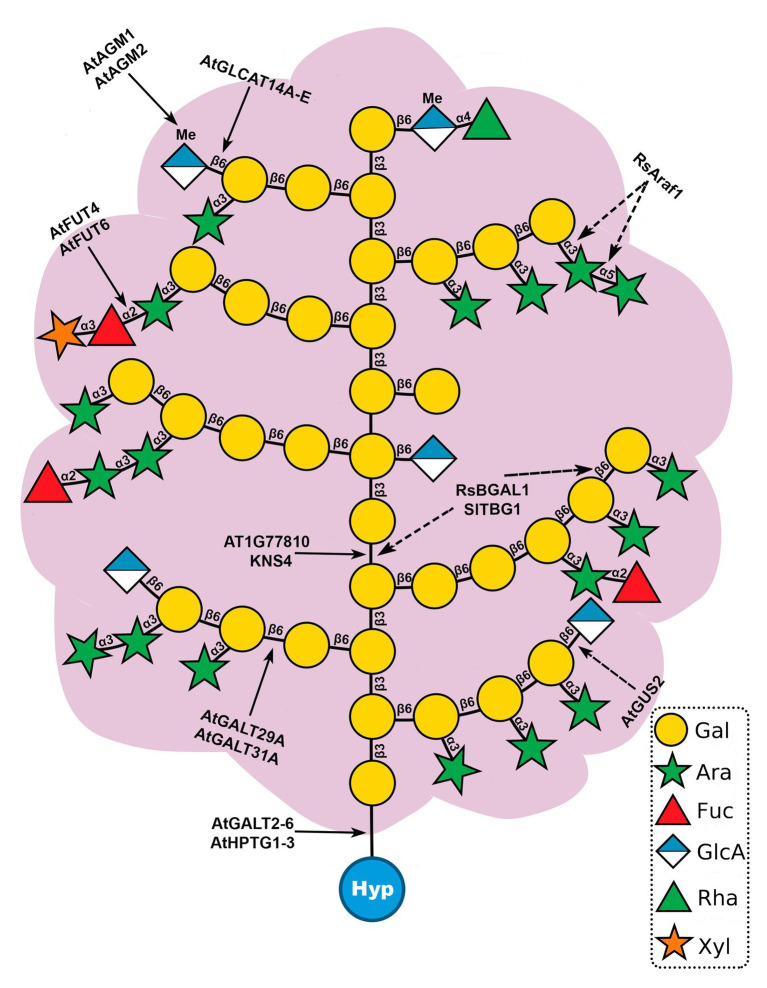
Model structure of type II arabinogalactan polysaccharides (AGs) and sites of action of known glycosyltransferases (GTs) and glycoside hydrolases (GHs) acting on AGPs. Type II AGs are O-glycosidically linked to hydroxyproline (Hyp) and consist of a β-(1→3)-linked backbone of galactose (Gal) with β-(1→6)-galactan side chains. Further modifications involve the addition of arabinose (Ara), fucose (Fuc), rhamnose (Rha), glucuronic acid (GlcA), 4-O-methylglucuronosyl (4-O-MeGlcA), and xylose (Xyl). Solid line arrows (left) represent sites of action of GTs and dotted line arrows (right) indicate sites of action of GHs. This structure is based on AGs analyzed from *Arabidopsis* leaves (Tryfona et al., 2012).

AG glycans have a complex and heterogenous structure that changes throughout development (Tryfona et al., 2014). AG polysaccharides may affect cell surface protein trafficking and stability, act as chaperoning polysaccharides or Ca^2+^ chelators and generate signaling molecules (Borner et al., 2003; Lamport and Várnai, 2013; Tryfona et al., 2014). In AGPs, type II AGs are O-glycosidically linked to Hyp residues (Ellis et al., 2010; Figure 2A). The structure of type II AGs is not fully resolved but it seems to consist of a β-(1→3)-linked D-galactopyranosyl (Gal*p*) backbone substituted at O6 positions by side-chains of β-(1→6)-linked D-Gal*p*, which are further decorated with α-(1→3)-l-arabinofuranosyl residues (Ara*f*) and less frequently with other sugars, such as GlcA, Rha, Fuc, and Xyl (Clarke et al., 1979; Gaspar et al., 2001; Showalter, 2001; Tan et al., 2004, 2010; Tryfona et al., 2010, 2012; Figure 3). The β-1,6-galactan side chains often terminate with β-1,6-GlcA or 4-O-Me-GlcA, giving the molecules an overall negative charge (Ellis et al., 2010; Tryfona et al., 2012; Temple et al., 2019). Two other types of AG polysaccharides have been described in plants (Clarke et al., 1979; Hijazi et al., 2014). Type I AGs are formed by a linear chain of β-(1→4)-linked D-Gal*p* with lateral chains of α-Ara*f* and β-(1→4)-linked D-Gal*p* (Clarke et al., 1979; Hijazi et al., 2014). Leonard et al. (2005) defined the *Artemisia vulgaris* Art v 1 polysaccharide, formed by a short linear β-(1→6)-linked D-Gal*p* chain, which contains Gal and Ara residues and large branched Ara chains, as a new AG type, the type III AG. However, this structure is inconsistent with the finding that it binds to β-Yariv, as chains of β-(1→3)-linked D-Gal*p*_7_ are required for β-Yariv binding (Kitazawa et al., 2013). Therefore, this structure may need to be revised.

Three models have been proposed for the molecular structure of AGPs: the wattle-blossom, the twisted hairy rope, and the necklace (Fincher et al., 1983; Qi et al., 1991; Du et al., 1996; Lamport et al., 2014). The wattle blossom model predicts globular units of polysaccharide chains anchored to a protein core of a spheroidal molecule, and the twisted hairy rope model foresees an alignment of AG chains along the protein backbone, whereas the recent necklace model compares AGP structure to an ancient gold necklace from Afghanistan with pendant glycomodules. Detailed analysis of different AGPs will determine the number, length, and sequence of polysaccharide chains, allowing the improvement of the existing models.

AGPs may be connected to other cell wall components such as pectins and hemicelluloses (Tan et al., 2013). Keegstra et al. (1973) hypothesized that type II AGs could be linked to rhamnogalacturonan (RGI) by the Rha residues. This linkage has been demonstrated in the complex arabinoxylan pectin arabinogalactan protein 1 (APAP1). This complex also has a link between arabinoxylan and an arabinose residue in the type II AGs (Tan et al., 2013). A FLA, SALT-OVERLY SENSITIVE 5 (SOS5) was proposed to mediate seed coat mucilage adherence by interacting with pectins (Griffiths et al., 2014). These results support the hypothesis that AGPs may serve as cross-linkers in the cell wall and act as polysaccharide plasticizers (Lamport, 2001; Lamport et al., 2006). The binding of the Yariv reagent specifically to AGs supports the view that AGs bind specific glycans (Kitazawa et al., 2013).

#### Glycosyltransferases: Meet the Sugar Workers

Glycoproteins have a complex structure and, therefore, plants require the action of numerous GTs families to assist in their biosynthesis. AGP glycosylation is catalyzed by a large number of GTs (EC 2.4.x.y) in the secretory pathway. GTs are enzymes that catalyze glycosidic bond formation between a sugar moiety and a specific acceptor molecule (sugars, proteins, lipids, or small molecules), creating a diverse collection of oligosaccharides and glycoconjugates in nature (Hansen et al., 2012; Gloster, 2014). GTs have been classified into 111 families in the carbohydrate active enzymes (CAZy) database^1^ (Coutinho et al., 2003; Lombard et al., 2014). GTs are very specific and, thus, each different linkage may require a distinct GT. With AGP glycosylation, several GTs work together to regulate the density, length, and sequences of AG chains (Qu et al., 2008). Almost 500 putative GTs sequences have been identified in the *Arabidopsis* genome and classified into 42 different CAZy families (Coutinho et al., 2003). In order to understand the structure of AGPs, the discovery of GTs involved in the initiation and elongation of AG chains is a priority. GTs can be type I membrane proteins located in the ER, type II membrane proteins located in the Golgi or integral membrane proteins such as cellulose synthases (Hansen et al., 2009). GTs generally are localized in the Golgi and have a type II membrane protein topology with a short N-terminal fragment facing the cytosol, one helical transmembrane domain, and a hydrophilic C-terminal catalytic domain containing the active site attached to a flexible stem region facing the luminal side (Perrin et al., 2001; Hansen et al., 2009; Chou et al., 2012).

The biosynthesis of AGP glycans requires at least 10 functionally distinct enzymes, e.g., galactosyltransferases (GALTs), arabinosyltransferases, fucosyltransferases (FUTs), rhamnosyltransferases, xylosyltransferases, glucuronosyltransferases (GLCATs), and glucuronic acid methyltransferases. To date, 22 transferases responsible for AGP glycosylation have been identified in *Arabidopsis* (Tables 1 and 2): eight hydroxyproline-O-β-GALTs (Hyp-O-GALTs; AtGALT2, AtGALT3, AtGALT4, AtGALT5, and AtGALT6, Basu et al., 2013, 2015a,b; Showalter and Basu, 2016; AtHPTG1, AtHPTG2, and AtHPTG3, Ogawa-Ohnishi and Matsubayashi, 2015), two β-1,3-GALTs (At1g77810, Qu et al., 2008 and AtKSN4, Suzuki et al., 2017), two β-1,6-GALTs (AtGALT31A, Geshi et al., 2013 and AtGALT29A; Dilokpimol et al., 2014), five β-1,6-GLCATs (AtGLCAT14A, AtGLCAT14B, AtGLCAT14C, AtGLCAT14D, and AtGLCAT14E, Knoch et al., 2013; Dilokpimol et al., 2014; Lopez-Hernandez et al., 2020; Zhang et al., 2020), two α-1,2-FUTs (AtFUT4 and AtFUT6, Wu et al., 2010; Liang et al., 2013; Tryfona et al., 2014), a putative β-arabinosyltransferase (AtRAY1, Gille et al., 2013), and two glucuronic acid methyltransferases (AtAGM1 and AtAGM2, Temple et al., 2019; Figure 3). However, several enzymes remain to be identified, including α-arabinofuranosyltransferases, β-arabinopyranosyltransferases, α-rhamnosyltransferases, α-xylosyltransferases, an α-GALTs, and other β-GALTs, β-GLCATs, α-FUTs, and glucuronic acid methyltransferases.

**Table 1:** Information about the characterized transferases involved in the biosynthesis of AGP glycans in *Arabidopsis thaliana*.

CAZy GT family	Enzyme	Locus	Enzyme Activity	Subcellular localization	References
GT14	GLCAT14A	At5g39990	β-1,6-GLCAT	Golgi Unique subcellular compartments	Knoch et al., 2013; Dilokpimol and Geshi, 2014; Lopez-Hernandez et al., 2020; Zhang et al., 2020
	GLCAT14B	At5g15050		-	
	GLCAT14C	At2g37585		-	
	GLCAT14D	At3g24040		-	
	GLCAT14E	At3g15350		-	
GT29	GALT29A	At1g08280	β-1,6-GALT	Golgi Unique subcellular compartments	Dilokpimol et al., 2014
GT31	GALT2	At4g21060	Hyp-O-β-GALT	ER and Golgi	Basu et al., 2013, 2015a,b
	GALT3	At3g06440		Golgi	
	GALT4	At1g27120		Golgi	
	GALT5	At1g74800		Golgi	
	GALT6	At5g62620		Golgi	
	HPGT1	At5g53340		Golgi	Ogawa-Ohnishi and Matsubayashi, 2015
	HPGT2	At4g32120		Golgi	
	HPGT3	At2g25300		Golgi	
	At1g77810	At1g77810	β-1,3-GALT	Golgi	Qu et al., 2008
	KNS4	At1g33430		-	Suzuki et al., 2017
	GALT31A	At1g32930	β-1,6-GALT	Golgi Unique subcellular compartments	Geshi et al., 2013
GT37	FUT4	At2g15390	α-1,2-FUT	-	Wu et al., 2010; Liang et al., 2013; Tryfona et al., 2014
	FUT6	At1g14080		Golgi	
GT77	RAY1	At1g70630	β-arabinofuranosyltransferase	Golgi	Gille et al., 2013
-	AGM1	At1g27930	GlcA O-methyltransferase	Golgi	Temple et al., 2019
	AGM2	At1g67330		-	

**Table 2:** Information about the mutants of the characterized transferases involved in the biosynthesis of AGP glycans in *Arabidopsis thaliana*.

Genes	Mutants	Mutation Localization	Mutant Phenotypes	References
*GLCAT14A*	*glcat14a-1* (SALK_064313)	Exon	Reduced of GlcA substitution on AGPs (*glcat14a*, *glcat14a-2*, *glcat14b-1*, *glcat14d-1*, *glcat14e-1*, *glcat14aglcat14b*, *glcat14a-2/b-1*, *glcat14bglcat14c*, *glcat14aglcat14bglcat14c*, *glcat14a-2/b-1/d-1*, *glcat14a-2/b-1/d-2*, and *glcat14a-2/b-1/e-1*); increase of Gal and Ara in AGPs (*glcat14c*, *glcat14aglcat14b*, *glcat14bglcat14c*, and *glcat14aglcat14bglcat14c*); reduction of Ara in AGPs (*glcat14a-1* and *glcat14a-2*); reduced amount of glycosylated AGPs (*glcat14c*, *glcat14aglcat14b*, and *glcat14aglcat14bglcat14c*); reduction in Ca^2+^ binding capacity of AGPs (*glcat14b*, *glcat14c*, *glcat14aglcat14b*, *glcat14bglcat14c*, *glcat14aglcat14bglcat14c*, and *glcat14a-2/b-1/d-1*); enhanced cell elongation in seedlings (*glcat14a-1* and *glcat14a-2*); reduced seed coat mucilage (*glcat14b*, *glcat14c*, *glcat14aglcat14b*, *glcat14bglcat14c*, and *glcat14aglcat14bglcat14c*); delayed seed germination and reduced root hair length (*glcat14aglcat14b* and *glcat14aglcat14bglcat14c*); reduced trichome branching (*glcat14aglcat14b*, *glcat14a-2/b-1*, *glcat14aglcat14bglcat14c*, *glcat14a-2/b-1/d-1*, and *glcat14a-2/b-1/d-2*); shorter siliques and reduced seed production (*glcat14bglcat14c* and *glcat14aglcat14bglcat14c*); small and defective pollen that failed to germinate (*glcat14aglcat14b* and *glcat14aglcat14bglcat14c*); shorter inflorescence stems (*glcat14a-2/b-1* and *glcat14a-2/b-1/d-1*); smaller etiolated hypocotyls and altered [Ca^2+^]cyt signature on roots (*glcat14a-2/b-1/e-1*)	Knoch et al., 2013; Dilokpimol and Geshi, 2014; Lopez-Hernandez et al., 2020; Zhang et al., 2020
	*glcat14a-2* (SALK_043905)	Exon		
	*glcat14a* (CRISPR-Cas9)	Exon		
*GLCAT14B*	*glcat14b* (CRISPR-Cas9)	Exon		
	*glcat14b-1* (SALK_080923)	Exon		
*GLCAT14C*	*glcat14c* (CRISPR-Cas9)	Exon		
*GLCAT14D*	*glcat14d-1* (GK363F05.01)	Exon		
	*glcat14d-2* (GK508D01)	Intron		
*GLCAT14E*	*glcat14e-1* (SALK_022820)	Intron		
*GALT29A*	-	-	-	Dilokpimol et al., 2014
*GALT2*	*galt2-1* (SALK_117233)	Exon	Lower GALT activities and reduced β-Yariv-precipitable AGPs; root and pollen tubes sensitivity reduction to β-Yariv reagent; reduced growth and root tip swelling in response to salt and sucrose; reduced root hair length and density (*galt2*, *galt3*, *galt5*, and *galt2galt5*), seed production (*galt4* and *galt6*), and seed coat mucilage (*galt3*, *galt6*, and *galt2galt5*); premature senescence (*galt6*); and large number of rosette leaves, delayed flowering, and shorter siliques (*galt2galt5*)	Basu et al., 2013, 2015a,b
	*galt2-2* (SALK_141126)	Exon		
*GALT3*	*galt3-1* (SALK_085633)	Promoter		
	*galt3-2* (SALK_005178)	Promoter		
*GALT4*	*galt4-1* (SALK_136251)	Exon		
	*galt4-2* (SALK_131723)	Exon		
*GALT5*	*galt5-1* (SALK_105404)	5' UTR		
	*galt5-2* (SALK_115741)	Exon		
*GALT6*	*galt6-1* (SAIL_59_D08)	Exon		
	*galt6-2* (SAIL_70_B02)	Exon		
*HPGT1*	*hpgt1-1* (SALK_007547)	Intron	Longer lateral roots, longer and denser root hairs, shorter inflorescence stems, and shorter siliques (*hpgt2-1*, *hpgt3-1*, and *hpgt1hpgt2hpgt3*) and thicker roots, smaller rosette leaves, shorter petioles, and reduced fertility (*hpgt1hpgt2hpgt3*)	Ogawa-Ohnishi and Matsubayashi, 2015
*HPGT2*	*hpgt2-1* (SALK_070368)	Exon		
*HPGT3*	*hpgt3-1* (SALK_009405)	Exon		
*At1g77810*	-	-	-	Qu et al., 2008
*KNS4*	-	-	-	Suzuki et al., 2017
*GALT31A*	*galt31a* (FLAG_379B06)	Exon	Embryo development arrested at the globular stage	Geshi et al., 2013
*FUT4*	*fut4* (SAIL_284_B05)	Exon	Reduced root growth under salt stress (*fut4*, *fut6*, and *fut4fut6*) and lack of fucose in leaf and root AGPs (*fut4fut6*)	Wu et al., 2010; Liang et al., 2013; Tryfona et al., 2014
	*fut4-2* (SALK_125310)	Exon		
*FUT6*	*fut6* (SALK_099500)	Exon		
	*fut6-2* (SALK_078257)	Exon		
*RAY1*	*ray1-1* (SALK_053158)	Exon	Reduced level of arabinose in their AGPs in etiolated seedlings, roots and rosette leaves; reduced root growth; and reduced rosette and inflorescence size	Gille et al., 2013
	*ray1-2* (GK_001C09)	Exon		
*AGM1*	*agm1-1* (SALK_000253)	Exon	Reduction on GlcA methylation (*agm1*); absence of GlcA methylation (*agm1agm2*)	Temple et al., 2019
*AGM2*	*agm2-1* (GABI_054A04)	Exon		
	*agm2-2* (SALK_057182)	5' UTR		

#### Galactosyltransferases: The First Ones to Arrive

AGP glycosylation is initiated by the action of Hyp-O-GALTs that add the first Gal onto the hydroxyl group of Hyp residues in the protein backbone (Ogawa-Ohnishi and Matsubayashi, 2015; Showalter and Basu, 2016). This process allows the subsequent addition of different sugars by other GTs. The eight GALTs specific for Hyp identified in *Arabidopsis* belong to the CAZy GT31 family. AtGALT2, AtGALT3, AtGALT4, AtGALT5, and AtGALT6 encode a GALT domain as well as a GALECTIN domain, whereas AtHPGT1, AtHPGT2, and AtHPGT3 lack a GALECTIN domain (Showalter and Basu, 2016). Their activity was demonstrated by heterologous expression in *Pichia pastoris* (AtGALT2, AtGALT5; Basu et al., 2013, 2015b), in *Nicotiana tabacum* leaf epidermal cells (AtGALT2, AtGALT3, AtGALT4, AtGALT5, and AtGALT6; Basu et al., 2015a) and BY-2 cells (AtHPGT1, AtHPGT2, and AtHPGT3; Ogawa-Ohnishi and Matsubayashi, 2015). *galt2*, *galt3*, *galt4*, *galt5*, *galt6*, *galt2galt5*, and *hpgt1hpgt2hpgt3* mutants demonstrated lower GALT activities and reduced β-Yariv-precipitable AGPs compared to wild-type plants. AtGALT3-6 and AtHPGT1 transiently expressed in *N. tabacum* and *Arabidopsis* T87 protoplasts, respectively, localized to the Golgi, whereas AtGALT2 was found in both ER and Golgi when expressed in tobacco (Basu et al., 2013, 2015a,b; Ogawa-Ohnishi and Matsubayashi, 2015). These results may indicate that AGP glycosylation may start in the ER, but predominantly occurs in the Golgi (Basu et al., 2013, 2016). Nevertheless, further studies are required to explore this hypothesis.

Some physiological phenotypes were revealed under normal growth conditions in the GALT mutants: *galt2*, *galt3*, *galt4*, *galt5*, and *galt2galt5* displayed reduced root hair length and density, *galt4* and *galt6* showed reduced seed production, *galt3*, *galt6*, and *galt2galt5* presented reduced seed coat mucilage and *galt6* revealed accelerated leaf senescence. Additionally, all GALT mutants (*galt2*, *galt3*, *galt4*, *galt5*, *galt6*, and *galt2galt5*) roots and pollen tubes exhibited less sensitivity to β-Yariv reagent, and root growth and root tip swelling were impaired under salt stress and in elevated levels of sucrose. *galt2galt5* displayed a large number of rosette leaves, delayed flowering time, reduced silique length, and plant height (Basu et al., 2015a,b). The *galt2galt5* double mutant phenocopies the root swelling, the reduced seed coat mucilage, and the reduced cellulose phenotypes of *sos5* and *fei1fei2*, a double mutant of two cell wall-associated leucine-rich repeat receptor-like kinases, and *sos5fei1fei2*, indicating that the carbohydrate moiety of SOS5 is important for signaling in the cell (Basu et al., 2015b). The analysis of the quintuple mutant *galt2galt5sos5fei1fei2* showed that these five genes act in a single and linear genetic pathway, and it was hypothesized that SOS5 glycosylation by GALT2 and GALT5 was required for its function in the SOS5/FEI1-FEI2 signaling pathway (Basu et al., 2016). In addition, HPGT mutants exhibited several pleiotropic phenotypes such as longer lateral roots, increased root hair length, and density, shorter inflorescence stems, shorter siliques (*hpgt2hpgt3* and *hpgt1hpgt2hpgt3*), thicker primary roots, smaller rosette leaves, shorter petioles, and reduced fertility in the lower portion of the inflorescence (*hpgt1hpgt2hpgt3*; Ogawa-Ohnishi and Matsubayashi, 2015).

Four additional GALTs were identified. At1g77810 encodes a β-1,3-GALT that belongs to the GT31 family and likely functions in β-1,3-galactan backbone synthesis. This Golgi membrane-located enzyme was demonstrated to add Gal to a synthetic β-1,3-Gal disaccharide using heterologous expression in COS cells (Qu et al., 2008). AtKSN4 (KAONASHI4) is also a member of GT31 family. Heterologous expression of AtKSN4 in *Nicotiana benthamiana* showed β-1,3-GALT activity on AG glycans from AGPs and pectins. *ksn4* mutants present an abnormality in the exine layer of developing microspores. Immunolabeling showed that *ksn4* mutants have reduced AGP content in the primexine of developing microspores. Furthermore, *ksn4* mutants exhibit pollen aggregation and reduced fertility (shorter fruit lengths and lower seed set compared to wild type; Suzuki et al., 2017). Another GT31 member, AtGALT31A, is involved in elongation of β-1,6-galactan side chains. Its activity was demonstrated by heterologous expression in *Escherichia coli* and *N. benthamiana*. AtGALT31A accumulates in the Golgi apparatus and unidentified organelles in *N. benthamiana*. A mutation in this gene showed an abnormal asymmetric cell division in the hypophysis causing the arrest of embryo development at the globular stage. This phenotype reveals the importance of AG glycans in embryo development (Geshi et al., 2013). AtGALT29A co-expresses with AtGALT31A and AtGLCAT14A. AtGALT29A resides in the GT29 family and this enzyme recombinantly expressed in *N. benthamiana* possesses β-1,6-GALT elongation and branch initiation activities. Dilokpimol et al. (2014) showed that AtGALT29A was localized in the Golgi in *N. benthamiana*, where it interacted with AtGALT31A, as indicated by Förster resonance energy transfer. AtGALT31A was also targeted in tobacco to uncharacterized small compartments, which are not part of the trans-Golgi network, cis-Golgi network, or endosomes and that colocalized with EXO70E2, a marker for exocyst-positive organelles that mediate an unconventional protein secretory pathway in plants (Poulsen et al., 2014). Moreover, the enzyme complex containing AtGALT31A and AtGALT29A exhibited enhanced β-1,6-GALT activity when compared to AtGALT29 alone.

##### Glucuronosyltransferases: Adding the Special Sugar

AtGLCAT14A, AtGLCAT14B, AtGLCAT14C AtGLCAT14D, and AtGLCAT14E are glucuronosyltransferases that belong to the GT14 family and add glucuronic acid to β-1,6‐ and β-1,3-galactose chains of AGPs. The activity of AtGLCAT14A, AtGLCAT14B, and AtGLCAT14C enzymes was confirmed by recombinant expression in *P. pastoris* and *in vitro* enzyme assays (Knoch et al., 2013; Dilokpimol and Geshi, 2014). AtGLCAT14A was transiently expressed in *N. benthamiana* and localized in the Golgi apparatus. AtGLCAT14A is co-localized with AtGALT31A in the Golgi and in uncharacterized small compartments, but the two enzymes do not interact (Knoch et al., 2013).

AtGLCAT14 mutants have defective synthesis of AGs. *glcat14a*, *glcat14b*, *glcat14d*, *glcat14e*, *glcat14aglcat14b*, *glcat14bglcat14c*, *glcat14aglcat14bglcat14c*, *glcat14aglcat14bglcat14d*, and *glcat14aglcat14bglcat14e* mutants have reduced the content of GlcA on AGPs when compared to wild type (Knoch et al., 2013; Lopez-Hernandez et al., 2020; Zhang et al., 2020). Although this suggests some redundancy between these enzymes, the mutants show some preferential changes in GlcA on specific branch lengths of AG and, therefore, may have roles glucuronidating different parts of the AG or different AGPs (Lopez-Hernandez et al., 2020). An increase of Gal and Ara was detected in AG extracts of *glcat14c*, *glcat14aglcat14b*, *glcat14bglcat14c*, and *glcat14aglcat14bglcat14c* (Zhang et al., 2020). However, in *glcat14a*, an increase of Gal and a reduction of Ara were detected. The increase of galactosylation may result from the increase of O6 acceptor sites, which are shared by AtGLCAT14A and GALTs and, thus, the addition of GlcA may terminate Gal chain extension (Knoch et al., 2013). Indeed, the amount of glycosylated AGPs was increased in the mutants *glcat14c*, *glcat14aglcat14b*, and *glcat14aglcat14bglcat14c*, which is a result of the loss of GlcA residues, allowing for the elongation of the branched β-(1,3)‐ and β-(1,6)-galactans. On the other hand, Lopez-Hernandez et al. (2020) did not find evidence for an increase in galactan side chain length in the *glcat14* mutants.

AtGLCAT14 mutants present several phenotypes. *glcat14a* knockout mutants showed enhanced cell elongation during seedling growth (Knoch et al., 2013), *glcat14b*, *glcat14c*, *glcat14aglcat14b*, *glcat14bglcat14c*, and *glcat14aglcat14bglcat14c* presented reduced seed coat mucilage, *glcat14aglcat14b* and *glcat14aglcat14bglcat14c* exhibited delayed seed germination, reduced root hair length, *glcat14aglcat14b*, *glcat14aglcat14bglcat14c*, and *glcat14aglcat14bglcat14d* reduced trichome branching, *glcat14aglcat14b* and *glcat14aglcat14bglcat14d* presented shorter inflorescences, *glcat14bglcat14c* and *glcat14aglcat14bglcat14c* presented reduced silique length and seed set, and *glcat14aglcat14b* and *glcat14aglcat14bglcat14c* displayed a significant percentage of small and defective pollen that failed to germinate (Lopez-Hernandez et al., 2020; Zhang et al., 2020). *glcat14aglcat14bglcat14e* triple mutant plants had severely limited seedling growth and were sterile (Lopez-Hernandez et al., 2020).

In addition, *glcat14b*, *glcat14c*, *glcat14aglcat14b*, *glcat14bglcat14c*, *glcat14aglcat14bglcat14c*, and *glcat14aglcat14bglcat14d* showed a reduction in Ca^2+^ binding in AGP extracts compared to wild type (Lopez-Hernandez et al., 2020; Zhang et al., 2020), consistent with the model proposed by Lamport and Várnai (2013) in which AGPs can bind and store Ca^2+^ through GlcA in a reversible and pH-dependent way at the plasma membrane. It was demonstrated that *in vitro* AGPs could hold Ca^2+^ in a pH range of 4–5 and as the pH was lowered Ca^2+^ was released. AGPs fully released Ca^2+^ at pH 2.5 (Lamport and Várnai, 2013). This bound Ca^2+^ may be important for intracellular signaling. Indeed, many of the plant developmental phenotypes in *glcat14* mutants can be suppressed by raising the Ca^2+^ concentration in the growth medium (Lopez-Hernandez et al., 2020). Intracellular Ca^2+^ signals were disrupted in the *glcat14aglcat14bglcat14e* mutant plants, which showed altered movement of the Ca^2+^ signal through the roots. Thus, GLCATs may play an important role in Ca^2+^ signaling as they determine the presence of GlcA on AGPs (Dilokpimol and Geshi, 2014).

##### Glucuronic Acid Methyltransferases: Changing GlcA

In type II AGs from *Arabidopsis*, most of the GlcA substituted is 4-O-methylglucuronosyl (4-O-Me-GlcA; Tryfona et al., 2012). Recently, two GlcA-specific methyltransferases have been identified in *Arabidopsis* (Temple et al., 2019). Arabinogalactan methyltransferases 1 (AGM1) and 2 (AGM2), two family members of DUF579 family, have GlcA-O-methylation activity on AGPs. AGM1 fused to GFP localized in the Golgi apparatus when transiently expressed in tobacco leaves. An *agm1* mutant showed reduced methylation of GlcA on root AGPs and in the double mutant *agm1agm2* there was no AG GlcA methylation of root AGPs. AGM1 and AGM2 are involved in methylation of GlcA of AG in root AGPs. An *agm1agm2* double mutant did not exhibit a growth or fertility phenotype, showing that GlcA methylation is not essential for viability. The biological role of GlcA methylation modification on AGPs is still unclear (Temple et al., 2019). However, it has been shown that the methyl group on GlcA is essential for the effectiveness of a signaling molecule in pollen tube guidance (Mizukami et al., 2016). In addition, 4-O-methylation of GlcA may change the Ca^2+^ binding affinity to GlcA thus modulating the calcium release response to pH (Lamport and Várnai, 2013). Moreover, the addition of the methyl group to GlcA prevents the addition of 4-linked sugars, such as Rha, and the extension of 4-linked side chains to the GlcA of AG as seen in APAP1 (Tan et al., 2013; Temple et al., 2019).

##### Fucosyltransferases: Continuing the Hard Work

AtFUT4 and AtFUT6, two AGP-α-(1,2)-FUT of AGPs, belong to the plant GT37 CAZy family. Their enzymatic activities were demonstrated by heterologous expression in *N. tabacum* BY2 cells (Wu et al., 2010). Tobacco BY2 cells contain nonfucosylated AGPs but heterologous expression of these genes resulted in fucosylated AGPs (Wu et al., 2010). Knock-out mutants (*fut4*, *fut6*, and *fut4fut6*) grown under salt stress showed reduced root growth (Liang et al., 2013; Tryfona et al., 2014). This conditional phenotype reveals the importance of this sugar in root growth and salt sensitivity. Fuc was absent in *fut4* leaf AGPs, whereas the *fut4fut6* double mutant lacks fucose in both leaf and root AGPs (Liang et al., 2013; Tryfona et al., 2014). As *AtFUT4* is expressed in roots and leaves while *AtFUT6* is expressed mostly in roots, it is likely that AtFUT4 and AtFUT6 are both responsible for AGP fucosylation in roots while AtFUT4 is also responsible for fucosylation in leaves (Wu et al., 2010; Liang et al., 2013; Tryfona et al., 2014). The *fut4* and *fut6* single mutants had reduced Fuc content in root AGPs. The *fut6* mutant was not stained by eel lectin that binds specifically to terminal α-L-Fuc, indicating that AtFUT6 likely adds terminal Fuc residues to AG polysaccharides (Liang et al., 2013). AtFUT6-GFP was transiently expressed in tobacco leaves and localized to the Golgi apparatus (Wu et al., 2010).

##### Arabinosyltransferases: The Ghost Workers

Finally, *ray1* mutants showed a reduced level of Ara in their AGPs in etiolated seedlings, roots, and rosette leaves of *Arabidopsis*, leading to its name REDUCED ARABINOSE YARIV 1. Knockout mutants also exhibit reduced root growth, reduced rosette diameter, and delayed development of the inflorescence. AtRAY1 belongs to the GT77 family, and its heterologous expression in *N. benthamiana* demonstrated β-arabinofuranosyltransferase activity (Gille et al., 2013). However, only α-1,3-linked Ara, and not β-1,3-linked Ara, has been reported in AGPs so it remains unclear whether and how RAY1 functions in the biosynthesis of AGPs glycans (Showalter and Basu, 2016). There are no published candidates for the α-arabinofuranosyltransferases that transfer the main arabinose decoration on AGs.

##### Glycoside Hydrolases: Cutting All the Sugar

GHs (EC 3.2.1.x) hydrolyze the glycosidic bond between two sugars and are likely important for the metabolism of AGPs (Henrissat and Davies, 1997; Knoch et al., 2014). GHs are classified into 167 families in the CAZy database (Coutinho et al., 2003; Lombard et al., 2014). Hydrolysis of AGP glycans requires several GHs, such as β-galactosidases, β-galactanases, α-arabinofuranosidases, β-arabinopyranosidases, β-glucuronidases, α-fucosidases, α-rhamnosidases, and β-xylosidases. These enzymes can be a useful tool to analyze AG sugars decorating AGPs (Knoch et al., 2014).

So far, five GHs of AGPs have been reported in plants (Figure 3). RsBGAL1, a β-galactosidase from *Raphanus sativus*, resides in the GH35 family and the protein when heterologously expressed in *P. pastoris* hydrolyzed β-(1,3)‐ and β-(1,6)-galactosyl residues (Kotake et al., 2005). RsAraf1 from *R. sativus* immature seeds encodes an α-arabinofuranosidase in the GH3 family. When expressed in *Arabidopsis*, RsAraf1 hydrolyzed α-arabinofuranosyl residues of AGPs (Kotake et al., 2006). AtGUS2 is a β-glucuronidase that belongs to the GH79 family. Knockout mutants showed increased levels of GlcA while plants over-expressing AtGUS2 showed the opposite phenotype, no GlcA in seedlings AGP fractions and increased cell elongation in seedlings, a similar phenotype of *atglcat14a* (Eudes et al., 2008). SlTBG1, a tomato (*Solanum lycopersicum*) β-galactosidase belonging to the GH35 family, has activity against β-(1,3)-linkages and β-(1,6)-linkages in a galactooligosaccharide, which may be involved in the hydrolysis of AGs from AGPs (Eda et al., 2014). AtAPSE (β-l-ARAPASE) is a β-l-arabinopyranosidase in the GH27 family. In an *apse* mutant, the amount of β-l-Ara*p* residues of AGPs was higher compared to wild type, confirming its activity (Imaizumi et al., 2017).

### AGPs in the Extracellular Space: Facing the Challenge

O-glycosylated GPI-anchored AGPs are transferred to the extracellular space, or more precisely to the cell surface apoplast, through the endomembrane system *via* post-Golgi vesicles, where they remain temporarily attached to the outer face of the plasma membrane (Schultz et al., 1998; Showalter, 2001). In response to intercellular or intracellular signals, GPI-anchored AGPs can be released from the plasma membrane by phosphatidylinositol-specific phospholipases that removes inositol from the diacylglycerol moiety (Schultz et al., 1998; Oxley and Bacic, 1999; Gillmor et al., 2005; Desnoyer and Palanivelu, 2020; Figure 2B). Up to the present, only one inositol phospholipid-specific phospholipase C (PI-PLC) has been identified, partially purified and characterized in plants (Butikofer and Brodbeck, 1993). Several GPI-anchored proteins such as FLAs, COBRA-like, and receptor-like proteins are released by phospholipases in *Arabidopsis* (Borner et al., 2003). This process may serve to regulate the amount of these glycoproteins in the plasma membrane and control the release of soluble AGPs into the extracellular matrix, where they may act as soluble signals for neighboring cells (Schultz et al., 1998). Nevertheless, some AGP members are not GPI-anchored. The sugars may be cleaved by GHs and released into the extracellular medium, where they may function as signaling molecules binding to specific receptors and triggering signaling pathways (Showalter, 2001; Figure 2B). Furthermore, AGPs may also be processed by proteolytic activities (Fincher et al., 1983; Faye et al., 2005) or transported by endocytotic multivesicular bodies to the vacuole, where they are degraded (Herman and Lamb, 1992; Šamaj et al., 2000).

## AGP Research Overview and Future Directions

In this review, information on AGP biosynthesis accumulated in the last three decades was presented. Much has been accomplished, but there is even more to be done. With the sequencing of several plant genomes, bioinformatics opened doors to identify several candidate genes involved in AGP biosynthesis. However, a huge amount of data is generated and much of it is waiting for further analysis. A major challenge will be to conduct the more laborious biochemical analyses to test and support the findings from bioinformatic and genomic analyses, which moves at such a rapid pace.

This review has largely focused on AGP glycosylation given the cumulative supporting information of the importance of the 90% sugar moiety of AGPs. There is no doubt that AGPs play a range of important functions in plants. The new challenge is to define how AGPs, after all the processes involved with their biosynthesis, act in the cells. Specifically, it will be important to elucidate the mechanism of action by which AGPs, and specifically their sugar epitopes, function. This will mean discovering the molecules, which interact with these sugar residues.

Several other important and stimulating questions remain to be answered: do the enzymes involved in AGP synthesis act on a specific single AGP, a subset of AGPs or all AGPs? How many GTs are involved in AGP glycosylation and what is the substrate specificity for each GT? Are the GTs involved in AGP biosynthesis associated with one another in enzyme complexes? The structure of AG is very variable, but is the pattern of glycosylation of AGPs cell/tissue-specific, stage-specific, or AGP-specific? What is the precise glycan structure of each AGP? Are the different patterns of glycosylation determined by the diverse amino acid sequences of AGPs? What implications do the different sugar compositions have in AGP function? Do AGPs follow a conventional or unconventional protein secretory pathway? Which AGPs localize in the plasma membrane? How does AGP turnover occur? These major challenges will be a stimulus for new future research developments on AGPs through the next 30 years.

## Author Contributions

JS organized and wrote the manuscript. RF assisted in writing the manuscript. SC proposed and corrected the manuscript. PD and AMS assisted in writing and revising the manuscript. All authors contributed to the article and approved the submitted version.

### Conflict of Interest

The authors declare that the research was conducted in the absence of any commercial or financial relationships that could be construed as a potential conflict of interest.

## Footnotes

^1^http://www.cazy.org
